# Bacillus Aerogenes Capsulatus (Welch) in Children’s Faeces

**Published:** 1915-02

**Authors:** 


**Bacillus Aerogenes Capsulatus (Welch) in Children's Faeces.**

J. H. M. Knox, Jr., and W. W. Ford of Johns Hopkins, Bulletin January, 1915, found this germ in all of 18 cases of minor digestive disturbances, in half at the first examination. On the other hand, it was absent in 15 to 20 tests each, in six out of seven breast-fed infants. The single positive finding was possibly due to contamination. They conclude that this germ is part of the normal flora of infants' intestines, unless breast fed.

